# The Hubble Constant

**DOI:** 10.1007/lrr-2015-2

**Published:** 2015-09-24

**Authors:** Neal Jackson

**Affiliations:** Jodrell Bank Centre for Astrophysics School of Physics and Astronomy, University of Manchester, Turing Building, Manchester, M13 9PL UK

**Keywords:** Cosmology, Hubble constant

## Abstract

I review the current state of determinations of the Hubble constant, which gives the length scale of the Universe by relating the expansion velocity of objects to their distance. There are two broad categories of measurements. The first uses individual astrophysical objects which have some property that allows their intrinsic luminosity or size to be determined, or allows the determination of their distance by geometric means. The second category comprises the use of all-sky cosmic microwave background, or correlations between large samples of galaxies, to determine information about the geometry of the Universe and hence the Hubble constant, typically in a combination with other cosmological parameters. Many, but not all, object-based measurements give *H*_0_ values of around 72–74 km s^−1^ Mpc^−1^, with typical errors of 2–3 km s^−1^ Mpc^−1^. This is in mild discrepancy with CMB-based measurements, in particular those from the *Planck* satellite, which give values of 67–68 km s^−1^ Mpc^−1^ and typical errors of 1–2 km s^−1^ Mpc^−1^. The size of the remaining systematics indicate that accuracy rather than precision is the remaining problem in a good determination of the Hubble constant. Whether a discrepancy exists, and whether new physics is needed to resolve it, depends on details of the systematics of the object-based methods, and also on the assumptions about other cosmological parameters and which datasets are combined in the case of the all-sky methods.

## Introduction

### A brief history

The last century saw an expansion in our view of the world from a static, Galaxy-sized Universe, whose constituents were stars and “nebulae” of unknown but possibly stellar origin, to the view that the observable Universe is in a state of expansion from an initial singularity over ten billion years ago, and contains approximately 100 billion galaxies. This paradigm shift was summarised in a famous debate between Shapley and Curtis in 1920; summaries of the views of each protagonist can be found in [43] and [195].

The historical background to this change in world view has been extensively discussed and whole books have been devoted to the subject of distance measurement in astronomy [176]. At the heart of the change was the conclusive proof that what we now know as external galaxies lay at huge distances, much greater than those between objects in our own Galaxy. The earliest such distance determinations included those of the galaxies NGC 6822 [93], M33 [94] and M31 [96], by Edwin Hubble.

As well as determining distances, Hubble also considered redshifts of spectral lines in galaxy spectra which had previously been measured by Slipher in a series of papers [197, 198]. If a spectral line of emitted wavelength λ_0_ is observed at a wavelength λ, the redshift *z* is defined as

1 $$z = \lambda/{\lambda _0} - 1.$$

**Figure 1:**
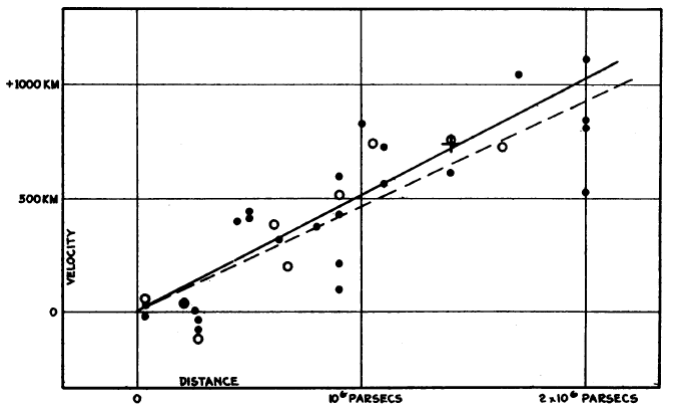
Hubble’s original diagram of distance to nearby galaxies, derived from measurements using Cepheid variables, against velocity, derived from redshift. The Hubble constant is the slope of this relation, and in this diagram is a factor of nearly 10 steeper than currently accepted values. Image reproduced from [95].

**Figure 2:**
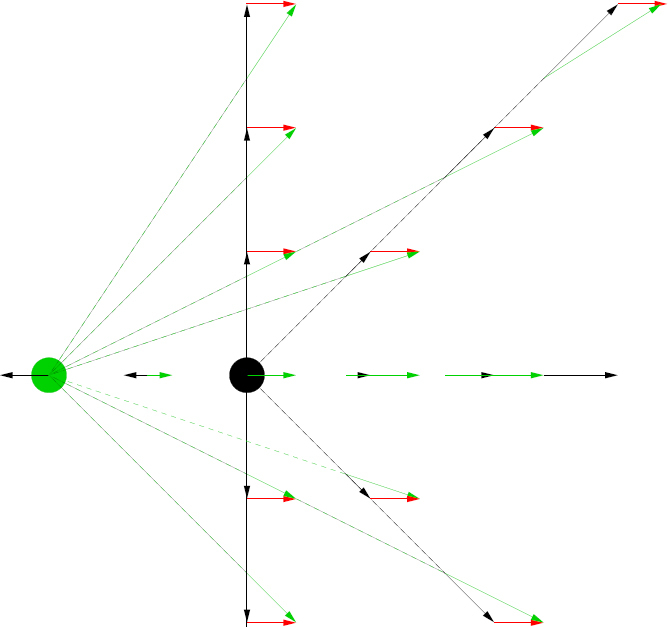
Illustration of the Hubble law. Galaxies at all points of the square grid are receding from the black galaxy at the centre, with velocities proportional to their distance away from it. From the point of view of the second, green, galaxy two grid points to the left, all velocities are modified by vector addition of its velocity relative to the black galaxy (red arrows). When this is done, velocities of galaxies as seen by the second galaxy are indicated by green arrows; they all appear to recede from this galaxy, again with a Hubble-law linear dependence of velocity on distance.

For nearby objects and assuming constant gravitational tidal field, the redshift may be thought of as corresponding to a recession velocity *v* which for nearby objects behaves in a way predicted by a simple Doppler formula,^1^ *v* = *cz*. Hubble showed that a relation existed between distance and redshift (see Figure 1); more distant galaxies recede faster, an observation which can naturally be explained if the Universe as a whole is expanding. The relation between the recession velocity and distance is linear in nearby objects, as it must be if the same dependence is to be observed from any other galaxy as it is from our own Galaxy (see Figure 2). The proportionality constant is the Hubble constant *H*_0_, where the subscript indicates a value as measured +now. Unless the Universe’s expansion does not accelerate or decelerate, the slope of the velocity-distance relation is different for observers at different epochs of the Universe. As well as the velocity corresponding to the universal expansion, a galaxy also has a “peculiar velocity”, typically of a few hundred kms^−1^, due to groups or clusters of galaxies in its vicinity. Peculiar velocities are a nuisance if determining the Hubble constant from relatively nearby objects for which they are comparable to the recession velocity. Once the distance is > 50 Mpc, the recession velocity is large enough for the error in *H*_0_ due to the peculiar velocity to be less than about 10%.

Recession velocities are very easy to measure; all we need is an object with an emission line and a spectrograph. Distances are very difficult. This is because in order to measure a distance, we need a standard candle (an object whose luminosity is known) or a standard ruler (an object whose length is known), and we then use apparent brightness or angular size to work out the distance. Good standard candles and standard rulers are in short supply because most such objects require that we understand their astrophysics well enough to work out what their luminosity or size actually is. Neither stars nor galaxies by themselves remotely approach the uniformity needed; even when selected by other, easily measurable properties such as colour, they range over orders of magnitude in luminosity and size for reasons that are astrophysically interesting but frustrating for distance measurement. The ideal *H*_0_ object, in fact, is one which involves as little astrophysics as possible.

Hubble originally used a class of stars known as Cepheid variables for his distance determinations. These are giant blue stars, the best known of which is *α*UMa, or Polaris. In most normal stars, a self-regulating mechanism exists in which any tendency for the star to expand or contract is quickly damped out. In a small range of temperature on the Hertzsprung-Russell (H-R) diagram, around 7000–8000 K, particularly at high luminosity,^2^ this does not happen and pulsations occur. These pulsations, the defining property of Cepheids, have a characteristic form, a steep rise followed by a gradual fall. They also have a period which is directly proportional to luminosity, because brighter stars are larger, and therefore take longer to pulsate. The period-luminosity relationship was discovered by Leavitt [123] by studying a sample of Cepheid variables in the Large Magellanic Cloud (LMC). Because these stars were known to be all at the same distance, their correlation of apparent magnitude with period therefore implied the *P-L* relationship.

The Hubble constant was originally measured as 500 km s^−1^ Mpc^−1^ [95] and its subsequent history was a more-or-less uniform revision downwards. In the early days this was caused by bias^3^ in the original samples [12], confusion between bright stars and HII regions in the original samples [97, 185] and differences between type I and II Cepheids^4^ [7]. In the second half of the last century, the subject was dominated by a lengthy dispute between investigators favouring values around 50 km s^−1^ Mpc^−1^ and those preferring higher values of 100 km s^−1^ Mpc^−1^. Most astronomers would now bet large amounts of money on the true value lying between these extremes, and this review is an attempt to explain why and also to try and evaluate the evidence for the best-guess current value. It is not an attempt to review the global history of *H*_0_ determinations, as this has been done many times, often by the original protagonists or their close collaborators. For an overall review of this process see, for example, [223] and [210]. Compilations of data and analysis of them are given by Huchra (http://cfa-www.harvard.edu/∼huchra/hubble), and Gott ([77], updated by [35]).^5^ Further reviews of the subject, with various different emphases and approaches, are given by [212, 68].

In summary, the ideal object for measuring the Hubble constant:

- Has a property which allows it to be treated as either as a standard candle or as a standard ruler
- Can be used independently of other calibrations (i.e., in a one-step process)
- Lies at a large enough distance (a few tens of Mpc or greater) that peculiar velocities are small compared to the recession velocity at that distance
- Involves as little astrophysics as possible, so that the distance determination does not depend on internal properties of the object
- Provides the Hubble constant independently of other cosmological parameters.

Many different methods are discussed in this review. We begin with one-step methods, and in particular with the use of megamasers in external galaxies — arguably the only method which satisfies all the above criteria. Two other one-step methods, gravitational lensing and Sunyaev-Zel’dovich measurements, which have significant contaminating astrophysical effects are also discussed. The review then discusses two other programmes: first, the Cepheid-based distance ladders, where the astrophysics is probably now well understood after decades of effort, but which are not one-step processes; and second, information from the CMB, an era where astrophysics is in the linear regime and therefore simpler, but where *H*_0_ is not determined independently of other cosmological parameters in a single experiment, without further assumptions.

### A little cosmology

The expanding Universe is a consequence, although not the only possible consequence, of general relativity coupled with the assumption that space is homogeneous (that is, it has the same average density of matter at all points at a given time) and isotropic (the same in all directions). In 1922, Friedman [72] showed that given that assumption, we can use the Einstein field equations of general relativity to write down the dynamics of the Universe using the following two equations, now known as the Friedman equations:

2 $${\dot a^2} - {1 \over 3}(8\pi G\rho + \Lambda){a^2} = - k{c^2},$$

3 $${{\ddot a} \over a} = - {4 \over 3}\pi G(\rho + 3p/{c^2}) + {1 \over 3}\Lambda.$$

Here *a* = *a*(*t*) is the scale factor of the Universe. It is fundamentally related to redshift, because the quantity (1 + *z*) is the ratio of the scale of the Universe now to the scale of the Universe at the time of emission of the light (*a*_0_/*a*). Λ is the cosmological constant, which appears in the field equation of general relativity as an extra term. It corresponds to a universal repulsion and was originally introduced by Einstein to coerce the Universe into being static. On Hubble’s discovery of the expansion of the Universe, he removed it, only for it to reappear seventy years later as a result of new data [157, 169] (see also [34, 235] for a review). *k* is a curvature term, and is −1, 0, or +1, according to whether the global geometry of the Universe is negatively curved, spatially flat, or positively curved. *ρ* is the density of the contents of the Universe, *p* is the pressure and dots represent time derivatives. For any particular component of the Universe, we need to specify an equation for the relation of pressure to density to solve these equations; for most components of interest such an equation is of the form *p* = *wρ*. Component densities vary with scale factor *a* as the Universe expands, and hence vary with time.

At any given time, we can define a Hubble parameter

4 $$H(t) = \dot a/a,$$

which is obviously related to the Hubble constant, because it is the ratio of an increase in scale factor to the scale factor itself. In fact, the Hubble constant *H*_0_ is just the value of *H* at the current time.^6^

If Λ = 0, we can derive the kinematics of the Universe quite simply from the first Friedman equation. For a spatially flat Universe *k* = 0, and we therefore have

5 $$\rho = {\rho _{\rm{c}}} \equiv {{3{H^2}} \over {8\pi G}},$$

where *ρ*_*c*_ is known as the critical density. For Universes whose densities are less than this critical density, *k* < 0 and space is negatively curved. For such Universes it is easy to see from the first Friedman equation that we require *ȧ* > 0, and therefore the Universe must carry on expanding for ever. For positively curved Universes (*k* > 0), the right hand side is negative, and we reach a point at which *ȧ* = 0. At this point the expansion will stop and thereafter go into reverse, leading eventually to a Big Crunch as *ȧ* becomes larger and more negative.

For the global history of the Universe in models with a cosmological constant, however, we need to consider the Λ term as providing an effective acceleration. If the cosmological constant is positive, the Universe is almost bound to expand forever, unless the matter density is very much greater than the energy density in cosmological constant and can collapse the Universe before the acceleration takes over. (A negative cosmological constant will always cause recollapse, but is not part of any currently likely world model). Carroll [34] provides further discussion of this point.

We can also introduce some dimensionless symbols for energy densities in the cosmological constant at the current time, ${\Omega _\Lambda} \equiv \Lambda/(3H_0^2)$, and in “curvature energy”, ${\Omega _k} \equiv - k{c^2}/H_0^2$ By rearranging the first Friedman equation we obtain

6 $${{{H^2}} \over {H_0^2}} = {\rho \over {{\rho _{\rm{c}}}}} - {\Omega _k}{a^{- 2}} + {\Omega _\Lambda}.$$

The density in a particular component of the Universe *X*, as a fraction of critical density, can be written as

7 $${\rho _X}/{\rho _{\rm{c}}} = {\Omega _X}{a^\alpha},$$

where the exponent *α* represents the dilution of the component as the Universe expands. It is related to the *w* parameter defined earlier by the equation *α* = −3(1 + *w*). For ordinary matter *α* = −3, and for radiation *α* = −4, because in addition to geometrical dilution as the universe expands, the energy of radiation decreases as the wavelength increases. The cosmological constant energy density remains the same no matter how the size of the Universe increases, hence for a cosmological constant we have *α* = 0 and *w* = −1. *w* = −1 is not the only possibility for producing acceleration, however. Any general class of “quintessence” models for which $w <  - {1 \over 3}$ will do; the case *w* < −1 is probably the most extreme and eventually results in the accelerating expansion becoming so dominant that all gravitational interactions become impossible due to the shrinking boundary of the observable Universe, finally resulting in all matter being torn apart in a “Big Rip” [32]. In current models Λ will become increasingly dominant in the dynamics of the Universe as it expands. Note that

8 $$\sum\limits_X {{\Omega _X}} + {\Omega _\Lambda} + {\Omega _k} = 1$$

by definition, because Ω_*k*_ = 0 implies a flat Universe in which the total energy density in matter together with the cosmological constant is equal to the critical density. Universes for which Ω_*k*_ is almost zero tend to evolve away from this point, so the observed near-flatness is a puzzle known as the “flatness problem”; the hypothesis of a period of rapid expansion known as inflation in the early history of the Universe predicts this near-flatness naturally. As well as a solution to the flatness problem, inflation is an attractive idea because it provides a natural explanation for the large-scale uniformity of the Universe in regions which would otherwise not be in causal contact with each other.

We finally obtain an equation for the variation of the Hubble parameter with time in terms of the Hubble constant (see, e.g., [155]),

9 $${H^2} = H_0^2({\Omega _\Lambda} + {\Omega _{\rm{m}}}{a^{- 3}} + {\Omega _{\rm{r}}}{a^{- 4}} + {\Omega _k}{a^{- 2}}),$$

where Ω_r_ represents the energy density in radiation and Ω_m_ the energy density in matter.

To obtain cosmological distances, we need to perform integrals of the form

10 $${D_C} = c\int {{{\rm{d}}z} \over {H(z)}},$$

where the right-hand side can be expressed as a “Hubble distance” *DH* ≡ *c*/*H*_0_, multiplied by an integral over dimensionless quantities such as the Ω terms. We can define a number of distances in cosmology, including the “comoving” distance *D*_*C*_ defined above. The most important for present purposes are the angular diameter distance *D*_A_ = *D*_C_/(1 + *z*), which relates the apparent angular size of an object to its proper size, and the luminosity distance *D*_L_ = (1 + *z*)^2^*D*_A_, which relates the observed flux of an object to its intrinsic luminosity. For currently popular models, the angular diameter distance increases to a maximum as *z* increases to a value of order 1, and decreases thereafter. Formulae for, and fuller explanations of, both distances are given by [87].

## One-Step Distance Methods

In this section, we examine the main methods for one-step Hubble constant determination using astrophysical objects, together with their associated problems and assess the observational situation with respect to each. Other methods have been proposed^7^ but do not yet have the observations needed to apply them.

### Megamaser cosmology

To determine the Hubble constant, measurements of distance are needed. In the nearby universe, the ideal object is one which is distant enough for peculiar velocities to be small — in practice around 50 Mpc — but for which a distance can be measured in one step and without a ladder of calibration involving other measurements in more nearby systems. Megamaser systems in external galaxies offer an opportunity to do this.

A megamaser system in a galaxy involves clumps of gas which are typically located ∼ 0.1 pc from the centre of the galaxy, close to the central supermassive black hole which is thought to lie at the centre of most if not all galaxies. These clumps radiate coherently in the water line at a frequency of approximately 22 GHz. This can be observed at the required milliarcsecond resolution scale using Very Long Baseline Interferometry (VLBI) techniques. With VLBI spectroscopy, the velocity of each individual clump can be measured accurately, and by repeated observations the movements of each clump can be followed and the acceleration determined. Assuming that the clumps are in Keplerian rotation, the radius of each clump from the central black hole can therefore be calculated, and the distance to the galaxy follows from knowledge of this radius together with the angular separation of the clump from the galaxy centre. The black-hole mass is also obtained as a by-product of the analysis. The analysis is not completely straightforward, as the disk is warped and viscous, with four parameters (eccentricity, position angle, periapsis angle and inclination) describing the global properties of the disk and four further parameters describing the properties of the warping [100]. In principle it is vulnerable to systematics involving the modelling parameters not adequately describing the disk, but such systematics can be simulated for plausible extra dynamical components [100] and are likely to be small.

The first maser system to be discovered in an external galaxy was that in the object NGC 4258. This galaxy has a shell of masers which are oriented almost edge-on [136, 79] and apparently in Keplerian rotation. Measurements of the distance to this galaxy have become steadily more accurate since the original work [84, 98, 100], although the distance of ∼ 7 Mpc to this object is not sufficient to avoid large (tens of percent) systematics due to peculiar velocities in any attempt to determine *H*_0_.

More recently, a systematic programme has been carried out to determine maser distances to other, more distant galaxies; the Megamaser Cosmology Project [167]. The first fruits of this programme include the measurement of the dynamics of the maser system in the galaxy UGC 3789, which have become steadily more accurate as the campaign has progressed [167, 25, 168]. A distance of 49.6±5.1 Mpc is determined, corresponding to *H*_0_ = 68.9±7.1 km s^−1^ Mpc^−1^ [168]; the error is dominated by the uncertainty in the likely peculiar velocity, which itself is derived from studies of the Tully-Fisher relation in nearby clusters [132]. Efforts are under way to find more megamasers to include in the sample, with success to date in the cases of NGC 6264 and Mrk 1419. Braatz et al. [24] and Kuo et al. [122] report preliminary results in the cases of the latter two objects, resulting in an overall determination of *H*_0_ = 68.0 ± 4.8 km s^−1^ Mpc^−1^ (68 ± 9 km s^−1^ Mpc^−1^ for NGC 6264). Tightening of the error bars as more megamasers are discovered, together with careful modelling, are likely to allow this project to make the cleanest determination of the Hubble constant within the next five years.

### Gravitational lenses

A general review of gravitational lensing is given by Wambsganss [233]; here we review the theory necessary for an understanding of the use of lenses in determining the Hubble constant. This determination, like the megamaser method, is a one-step process, although at a much greater distance. It is thus interesting both as a complementary determination and as an opportunity to determine the Hubble parameter as a function of redshift. It has the drawback of possessing one serious systematic error associated with contaminating astrophysics, namely the detailed mass model of the lens.

#### Basics of lensing

Light is bent by the action of a gravitational field. In the case where a galaxy lies close to the line of sight to a background quasar, the quasar’s light may travel along several different paths to the observer, resulting in more than one image.

The easiest way to visualise this is to begin with a zero-mass galaxy (which bends no light rays) acting as the lens, and considering all possible light paths from the quasar to the observer which have a bend in the lens plane. From the observer’s point of view, we can connect all paths which take the same time to reach the observer with a contour in the lens plane, which in this case is circular in shape. The image will form at the centre of the diagram, surrounded by circles representing increasing light travel times. This is of course an application of Fermat’s principle; images form at stationary points in the Fermat surface, in this case at the Fermat minimum. Put less technically, the light has taken a straight-line path^8^ between the source and observer.

**Figure 3:**
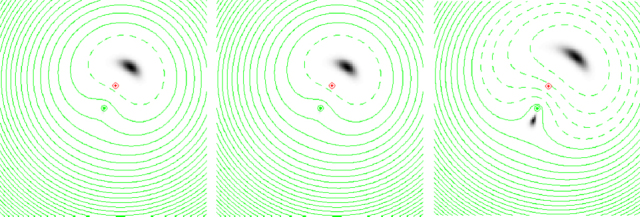
Illustration of a Fermat surface for a source (red symbol) close to the line of sight to a galaxy (green symbol). In each case the appearance of the images to the observer is shown by a greyscale, and the contours of the Fermat surface are given by green contours. Note that images form at stationary points of the surface defined by the contours. In the three panels, the mass of the galaxy, and thus the distortion of the Fermat surface, increases, resulting in an increasingly visible secondary image at the position of the saddle point. At the same time, the primary image moves further from the line of sight to the source. In each case the third image, at the position of the Fermat maximum, is too faint to see.

If we now allow the galaxy to have a steadily increasing mass, we introduce an extra time delay (known as the Shapiro delay) along light paths which pass through the lens plane close to the galaxy centre. This makes a distortion in the Fermat surface (Figure 3). At first, its only effect is to displace the Fermat minimum away from the distortion. Eventually, however, the distortion becomes big enough to produce a maximum at the position of the galaxy, together with a saddle point on the other side of the galaxy from the minimum. By Fermat’s principle, two further images will appear at these two stationary points in the Fermat surface. This is the basic three-image lens configuration, although in practice the central image at the Fermat maximum is highly demagnified and not usually seen.

If the lens is significantly elliptical and the lines of sight are well aligned, we can produce five images, consisting of four images around a ring alternating between maxima and saddle points, and a central, highly demagnified Fermat maximum. Both four-image and two-image systems (“quads” and “doubles”) are in fact seen in practice. The major use of lens systems is for determining mass distributions in the lens galaxy, since the positions and fluxes of the images carry information about the gravitational potential of the lens. Gravitational lensing has the advantage that its effects are independent of whether the matter is light or dark, so in principle the effects of both baryonic and non-baryonic matter can be probed.

#### Principles of time delays

**Figure 4:**
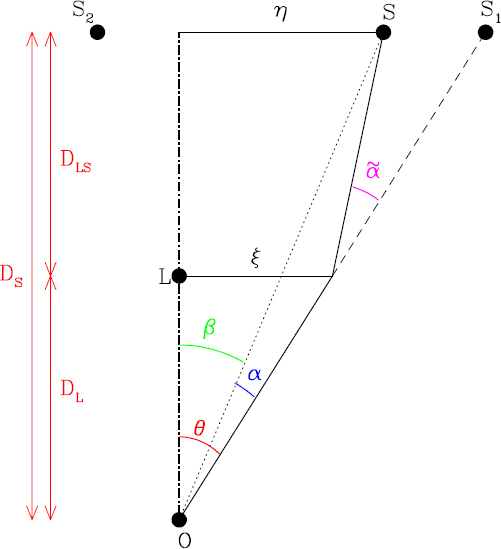
Basic geometry of a gravitational lens system. Image reproduced from [233]; copyright by the author.

Refsdal [166] pointed out that if the background source is variable, it is possible to measure an absolute distance within the system and therefore the Hubble constant. To see how this works, consider the light paths from the source to the observer corresponding to the individual lensed images. Although each is at a stationary point in the Fermat time delay surface, the absolute light travel time for each will generally be different, with one of the Fermat minima having the smallest travel time. Therefore, if the source brightens, this brightening will reach the observer at different times corresponding to the two different light paths. Measurement of the time delay corresponds to measuring the difference in the light travel times, each of which is individually given by

11 $$\tau = {{{D_{\rm{l}}}{D_{\rm{s}}}} \over {c{D_{{\rm{ls}}}}}}(1 + {z_{\rm{l}}})\left({{1 \over 2}{{(\theta - \beta)}^2} - \psi (\theta)} \right),$$

where *α*, *β* and *θ* are angles defined below in Figure 4, *D*_l_, *D*_s_ and *D*_ls_ are angular diameter distances also defined in Figure 4, *z*_l_ is the lens redshift, and *ψ*(*θ*) is a term representing the Shapiro delay of light passing through a gravitational field. Fermat’s principle corresponds to the requirement that ∇*τ* = 0. Once the differential time delays are known, we can then calculate the ratio of angular diameter distances which appears in the above equation. If the source and lens redshifts are known, *H*_0_ follows from Eqs. 9 and 10. The value derived depends on the geometric cosmological parameters Ω_*m*_ and Ω_Λ_, but this dependence is relatively weak. A handy rule of thumb which can be derived from this equation for the case of a 2-image lens, if we make the assumption that the matter distribution is isothermal^9^ and *H*_0_ = 70 km s^−1^ Mpc^−1^, is

12 $$\Delta \tau = (14\,{\rm{days}})\,(1 + {z_1})D\left({{{f - 1} \over {f + 1}}} \right){s^2},$$

where *z*_1_ is the lens redshift, *s* is the separation of the images (approximately twice the Einstein radius), *f* > 1 is the ratio of the fluxes and *D* is the value of *D*_s_*D*_l_/*D*_ls_ in Gpc. A larger time delay implies a correspondingly lower *H*_0_.

**Figure 5:**
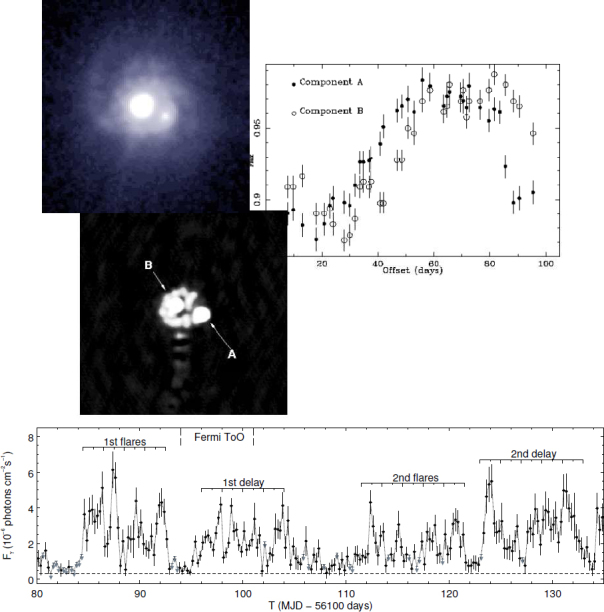
The lens system JVAS B0218+357. *Top right*: the measurement of time delay of about 10 days from asynchronous variations of the two lensed images [16]. The *upper left* panels show the HST/ACS image [241] on which can be seen the two images and the spiral lensing galaxy, and the radio MERLIN+VLA image [17] showing the two images together with an Einstein ring. The *bottom panel* shows the *γ*-ray lightcurve [37], in which, although the components are not resolved, the sharpness of the variations allows a time delay to be determined (at 11.46 ± 0.16 days, significantly greater than the radio time delay). Image reproduced with permission from [37]; copyright by AAS.

The first gravitational lens was discovered in 1979 [232] and monitoring programmes began soon afterwards to determine the time delay. This turned out to be a long process involving a dispute between proponents of a ∼ 400-day and a ∼ 550-day delay, and ended with a determination of 417 ± 2 days [121, 189]. Since that time, over 20 more time delays have been determined (see Table 1). In the early days, many of the time delays were measured at radio wavelengths by examination of those systems in which a radio-loud quasar was the multiply imaged source (see Figure 5). Recently, optically-measured delays have dominated, due to the fact that only a small optical telescope in a site with good seeing is needed for the photometric monitoring, whereas radio time delays require large amounts of time on long-baseline interferometers which do not exist in large numbers.^10^ A time delay using *γ*-rays has been determined for one lens [37] using correlated variations in a light-curve which contains emission from both images of the lens.

**Table 1:** Time delays, with 1-*σ* errors, from the literature. In some cases multiple delays have been measured in 4-image lens systems, and in this case each delay is given separately for the two components in brackets. An additional time delay for CLASS B1422+231 [151] probably requires verification, and a published time delay for Q0142-100 [120, 146] has large errors. Time delays for the CLASS and PKS objects have been obtained using radio interferometers, and the remainder using optical telescopes.

Lens system	Time delay [days]	Reference
CLASS 0218+357	10.5 ± 0.2	[16]
HE 0435-1-223	$14.4_{- 0.9}^{+ 0.8}({\rm{AD}})$	[116]
	7.8 ± 0.8 (BC)	also others [41]
SBS 0909+532	$45_{- 11}^{+ 1}({\rm{2}}\sigma)$	[229]
RX 0911+0551	146 ± 4	[86]
FBQ 0951+2635	16 ± 2	[103]
Q 0957+561	417 ± 3	[121]
SDSS 1001+5027	119.3 ± 3.3	[164]
SDSS 1004+4112	38.4 ± 2.0 (AB)	[65]
SDSS 1029+2623		[64]
HE 1104-185	161 ± 7	[140]
PG 1115+080	23.7 ± 3.4 (BC)	[188]
	9.4 ± 3.4 (AC)	
RX 1131-1231	$12.0_{- 1.3}^{+ 1.5}({\rm{AB}})$	[138]
	$9.6_{- 1.6}^{+ 2.0}({\rm{AC}})$	
	87 ± 8 (AD)	
		[217]
SDSS J1206+4332	111.3 ± 3	[57]
SBS 1520+530	130 ± 3	[30]
CLASS 1600+434	51 ± 2	[28]
	$47_{- 6}^{+ 5}$	[118]
CLASS 1608+656	$31.5_{- 1}^{+ 2}({\rm{AB}})$	[61]
	$36_{- 2}^{+ 1}({\rm{BC}})$	
	$77_{- 1}^{+ 2}({\rm{BD}})$	
SDSS 1650+4251	49.5 ± 1.9	[230]
PKS 1830-211	$26_{- 5}^{+ 4}$	[127]
WFI J2033-4723	35.5 ± 1.4 (AB)	[231]
HE 2149-2745	103 ± 12	[29]
HS 2209+1914	20.0 ± 5	[57]
Q 2237+0305	$2.7_{- 0.9}^{+ 0.5}({\rm{h}})$	[44]

#### The problem with lens time delays

Unlike local distance determinations (and even unlike cosmological probes which typically use more than one measurement), there is only one major systematic piece of astrophysics in the determination of *H*_0_ by lenses, but it is a very important one.^11^ This is the form of the potential in Eq. (11). If one parametrises the potential in the form of a power law in projected mass density versus radius, the index is −1 for an isothermal model. This index has a pretty direct degeneracy^12^ with the deduced length scale and therefore the Hubble constant; for a change of 0.1, the length scale changes by about 10%. The sense of the effect is that a steeper index, which corresponds to a more centrally concentrated mass distribution, decreases all the length scales and therefore implies a higher Hubble constant for a given time delay.

If an uncertainty in the slope of a power-law mass distribution were the only issue, then this could be constrained by lensing observables in the case where the source is extended, resulting in measurements of lensed structure at many different points in the lens plane [115]. This has been done, for example, using multiple radio sources [38], VLBI radio structure [239] and in many objects using lensed structure of background galaxies [21], although in this latter case *H*_0_ is not measurable because the background objects are not variable. The degeneracy between the Hubble constant and the mass model is more general than this, however [76]. The reason is that lensing observables give information about the derivatives of the Fermat surface; the positions of the images are determined by the first derivatives of the surface, and the fluxes by the second derivatives. For any given set of lensing observables, we can move the intrinsic source position, thus changing the Fermat surface, and then restore the observables to their original values by adjusting the mass model and thus returning the Fermat surface to its original configuration. It therefore follows that any given set of measurements of image positions and fluxes in a lens system is consistent with a number of different mass models, and therefore a number of different values of *H*_0_, because the source position cannot be determined. Therefore the assumption of a particular type of model, such as a power-law, itself constitutes a selection of a particular one out of a range of possible models [192], each of which would give a different *H*_0_. Modelling degeneracies arise not only from the mass distribution within the lens galaxy, but also from matter along the line of sight. These operate in the sense that, if a mass sheet is present which is not known about, the length scale obtained is too short and consequently the derived value of *H*_0_ is too high.

There are a number of approaches to this mass-degeneracy problem. The first is to use a non-parametric model for the projected mass distribution, imposing only a minimum number of physically-motivated requirements such as monotonicity, and thereby generate large numbers of mass models which are exactly consistent with the data. This was pioneered by Saha and Williams in a series of papers [179, 237, 180, 177] in which pixellated models of galaxy mass distributions were used. Although pixellated models are useful for exploring the space of allowed models, they do not break the essential degeneracy. Other priors may be used, however: in principle it should also be possible to reject some possible mass distributions on physical grounds, because we expect the mass profiles to contain a central stellar cusp and a more extended dark matter halo. Undisturbed dark matter haloes should have profiles similar to a Navarro, Frenk & White (NFW, [139]) form, but they may be modified by adiabatic contraction during the process of baryonic infall when the galaxy forms.

Second, it is possible to increase the reliability of individual lens mass models by gathering extra information which partially breaks the mass degeneracy. A major improvement is available by the use of stellar velocity dispersions [221, 220, 222, 119] measured in the lensing galaxy. As a standalone determinant of mass models in galaxies at *z* ∼ 0.5, typical of lens galaxies, such measurements are not very useful as they suffer from severe degeneracies with the structure of stellar orbits. However, the combination of lensing information (which gives a very accurate measurement of mass enclosed by the Einstein radius) and stellar dynamics (which gives, more or less, the mass enclosed within the effective radius of the stellar light) gives a measurement that in effect selects only some of the family of possible lens models which fit a given set of lensing observables. The method has large error bars, in part due to residual dependencies on the shape of stellar orbits, but also because these measurements are very difficult; each galaxy requires about one night of good seeing on a 10-m telescope. Nevertheless, this programme has the potential beneficial effect of reducing the dominant systematic error, despite the potential additional systematic from the assumptions about stellar orbits.

Third, we can remove problems associated with mass sheets associated with material extrinsic to the main lensing galaxy by measuring them using detailed studies of the environments of lens galaxies. Studies of lens groups [60, 106, 59, 137] show that neglecting matter along the line of sight typically has an effect of 10–20%, with matter close to the redshift of the lens contributing most. More recently, it has been shown that a combination of studies of number counts and redshifts of nearby objects to the main lens galaxy, coupled with comparisons to large numerical simulations of matter such as the Millenium Simulation, can reduce the errors associated with the environment to around 3–4% [78].

#### Time delay measurements

Table 1 shows the currently measured time delays, with references and comments. The addition of new measurements is now occurring at a much faster rate, due to the advent of more systematic dedicated monitoring programmes, in particular that of the COSMOGRAIL collaboration (e.g., [230, 231, 41, 164, 57]). Considerable patience is needed for these efforts in order to determine an unambiguous delay for any given object, given the contaminating effects of microlensing and also the unavoidable gaps in the monitoring schedule (at least for optical monitoring programmes) once per year as the objects move into the daytime. Derivation of time delays under these circumstances is not a trivial matter, and algorithms which can cope with these effects have been under continuous development for decades [156, 114, 88, 217] culminating in a blind analysis challenge [50].

#### Derivation of *H*_0_: Now, and the future

Initially, time delays were usually turned into Hubble constant values using assumptions about the mass model — usually that of a single, isothermal power law [119] — and with rudimentary modelling of the environment of the lens system as necessary. Early analyses of this type resulted in rather low values of the Hubble constant [112] for some systems, sometimes due to the steepness of the lens potential [221]. As the number of measured time delays expanded, combined analyses of multiple lens systems were conducted, often assuming parametric lens models [141] but also using Monte Carlo methods to account for quantities such as the presence of clusters around the main lens. These methods typically give values around 70 km s^−1^ Mpc^−1^ — e.g., (68 ± 6 ± 8) km s^−1^ Mpc^−1^ from Oguri (2007) [141], but with an uncomfortably greater spread between lens systems than would be expected on the basis of the formal errors. An alternative approach to composite modelling is to use non-parametric lens models, on the grounds that these may permit a wider range of mass distributions [177, 150] even though they also contain some level of prior assumptions. Saha et al. (2006) [177] used ten time-delay lenses for this purpose, and Paraficz et al. (2010) [150] extended the analysis to eighteen systems obtaining $66_{- 4}^{+ 6}$, with a further extension by Sereno & Paraficz (2014) [194] giving 66 ± 6 ± 4 (stat/syst) km s^−1^ Mpc^−1^.

In the last few years, concerted attempts have emerged to put together improved time-delay observations with systematic modelling. For two existing time-delay lenses (CLASS B1608+656 and RXJ 1131-1231) modelling has been undertaken [205, 206] using a combination of all of the previously described ingredients: stellar velocity dispersions to constrain the lens model and partly break the mass degeneracy, multi-band HST imaging to evaluate and model the extended light distribution of the lensed object, comparison with numerical simulations to gauge the likely contribution of the line of sight to the lensing potential, and the performance of the analysis blind (without sight of the consequences for *H*_0_ of any decision taken during the modelling). The results of the two lenses together, $75.2_{- 4.2}^{+ 4.4}$ and $73.1_{- 3.6}^{+ 2.4}$ in flat and open ΛCDM, respectively, are probably the most reliable determinations of *H*_0_ from lensing to date, even if they do not have the lowest formal error^13^.

In the immediate future, the most likely advances come from further analysis of existing time delay lenses, although the process of obtaining the data for good quality time delays and constraints on the mass model is not a quick process. A number of further developments will expedite the process. The first is the likely discovery of lenses on an industrial scale using the Large Synoptic Survey Telescope (LSST, [101]) and the *Euclid* satellite [4], together with time delays produced by high cadence monitoring. The second is the availability in a few years’ time of > 8-m class optical telescopes, which will ease the followup problem considerably. A third possibility which has been discussed in the past is the use of double source-plane lenses, in which two background objects, one of which is a quasar, are imaged by a single foreground object [74, 39]. Unfortunately, it appears [191] that even this additional set of constraints leave the mass degeneracy intact, although it remains to be seen whether dynamical information will help relatively more in these objects than in single-plane systems.

One potentially clean way to break mass model degeneracies is to discover a lensed type Ia supernova [142, 143]. The reason is that, as we have seen, the intrinsic brightness of SNe Ia can be determined from their lightcurve, and it can be shown that the resulting absolute magnification of the images can then be used to bypass the effective degeneracy between the Hubble constant and the radial mass slope. Oguri et al. [143] and also Bolton and Burles [20] discuss prospects for finding such objects; future surveys with the Large Synoptic Survey Telescope (LSST) are likely to uncover significant numbers of such events. The problem is likely to be the determination of the time delay, since nearly all such objects are subject to significant microlensing effects within the lensing galaxy which is likely to restrict the accuracy of the measurement [51].

### The Sunyaev-Zel’dovich effect

The basic principle of the Sunyaev-Zel’dovich (S-Z) method [203], including its use to determine the Hubble constant [196], is reviewed in detail in [18, 33]. It is based on the physics of hot (10^8^ K) gas in clusters, which emits X-rays by bremsstrahlung emission with a surface brightness given by the equation (see e.g., [18])

13 $${b_{\rm{X}}} = {1 \over {4\pi {{(1 + z)}^3}}}\int n_e^2{\Lambda _e}\,{\rm{d}}l,$$

where *n*_*e*_ is the electron density and Λ_*e*_ the spectral emissivity, which depends on the electron temperature.

**Figure 6:**
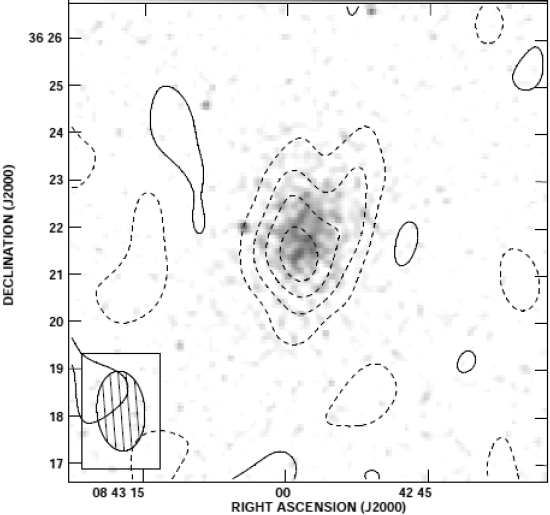
S-Z decrement observation of Abell 697 with the Ryle telescope in contours superimposed on the *ROSAT* image (grey-scale). Image reproduced with permission from [104]; copyright by RAS.

At the same time, the electrons of the hot gas in the cluster Compton upscatter photons from the CMB radiation. At radio frequencies below the peak of the Planck distribution, this causes a “hole” in radio emission as photons are removed from this spectral region and turned into higher-frequency photons (see Figure 6). The decrement is given by an optical-depth equation,

14 $$\Delta I(\nu) = {I_0}\int {{n_e}{\sigma _T}\Psi (\nu, {T_e})\,\,{\rm{d}}l,}$$

involving many of the same parameters and a function Ψ which depends on frequency and electron temperature. It follows that, if both *b*_X_ and Δ*I*(*x*) can be measured, we have two equations for the variables *n*_*e*_ and the integrated length *l*_∥_ through the cluster and can calculate both quantities. Finally, if we assume that the projected size *l*_⊥_ of the cluster on the sky is equal to *l*_∥_, we can then derive an angular diameter distance if we know the angular size of the cluster. The Hubble constant is then easy to calculate, given the redshift of the cluster.

Although in principle a clean, single-step method, in practice there are a number of possible difficulties. Firstly, the method involves two measurements, each with a list of possible errors. The X-ray determination carries a calibration uncertainty and an uncertainty due to absorption by neutral hydrogen along the line of sight. The radio observation, as well as the calibration, is subject to possible errors due to subtraction of radio sources within the cluster which are unrelated to the S-Z effect. Next, and probably most importantly, are the errors associated with the cluster modelling. In order to extract parameters such as electron temperature, we need to model the physics of the X-ray cluster. This is not as difficult as it sounds, because X-ray spectral information is usually available, and line ratio measurements give diagnostics of physical parameters. For this modelling the cluster is usually assumed to be in hydrostatic equilibrium, or a “beta-model” (a dependence of electron density with radius of the form $n(r) = {n_0}{(1 + {r^2}/r_{\rm{c}}^2)^{- 3\beta/2}}$ is assumed. Several recent works [190, 22] relax this assumption, instead constraining the profile of the cluster with available X-ray information, and the dependence of *H*_0_ on these details is often reassuringly small (< 10%). Finally, the cluster selection can be done carefully to avoid looking at prolate clusters along the long axis (for which *l*_⊥_ ≠ *l*_∥_) and therefore seeing more X-rays than one would predict. This can be done by avoiding clusters close to the flux limit of X-ray flux-limited samples, Reese et al. [165] estimate an overall random error budget of 20–30% for individual clusters. As in the case of gravitational lenses, the problem then becomes the relatively trivial one of making more measurements, provided there are no unforeseen systematics.

The cluster samples of the most recent S-Z determinations (see Table 2) are not independent in that different authors often observe the same clusters. The most recent work, that in [22] is larger than the others and gives a higher *H*_0_. It is worth noting, however, that if we draw subsamples from this work and compare the results with the other S-Z work, the *H*_0_ values from the subsamples are consistent. For example, the *H*_0_ derived from the data in [22] and modelling of the five clusters also considered in [104] is actually lower than the value of 66 km s^−1^ Mpc^−1^ in [104]. Within the smaller samples, the scatter is much lower than the quoted errors, partially due to the overlap in samples (three objects are common to more than one of the four smaller studies).

**Table 2:** Some recent measurements of *H*_0_ using the S-Z effect. Model types are *β* for the assumption of a *β*-model and H for a hydrostatic equilibrium model. Some of the studies target the same clusters, with three objects being common to more than one of the four smaller studies, The larger study [22] contains four of the objects from [104] and two from [190].

Reference	Number of clusters	Model type	*H*_0_ determination [km s^−1^ Mpc^−1^]
[22]	38	*β* + H	$76.9_{- 3.4 - 8.0}^{+ 3.9 + 10.0}$
[104]	5	*β*	$66_{- 10 - 8}^{+ 11 + 9}$
[228]	7	*β*	$67_{- 18 - 6}^{+ 30 + 15}$
[190]	3	H	69 ± 8
[131]	7	*β*	$66_{- 11 - 15}^{+ 14 + 15}$
[165]	18	*β*	$60_{- 4 - 18}^{+ 4 + 13}$

It therefore seems as though S-Z determinations of the Hubble constant are beginning to converge to a value of around 70 km s^−1^ Mpc^−1^, although the errors are still large, values in the low to mid-sixties are still consistent with the data and it is possible that some objects may have been observed but not used to derive a published *H*_0_ value. Even more than in the case of gravitational lenses, measurements of *H*_0_ from individual clusters are occasionally discrepant by factors of nearly two in either direction, and it would probably teach us interesting astrophysics to investigate these cases further.

### Gamma-ray propagation

High-energy *γ*-rays emitted by distant AGN are subject to interactions with ambient photons during their passage towards us, producing electron-positron pairs. The mean free path for this process varies with photon energy, being smaller at higher energies, and is generally a substantial fraction of the distance to the sources. The observed spectrum of *γ*-ray sources therefore shows a high-energy cutoff, whose characteristic energy decreases with increasing redshift. The expected cutoff, and its dependence on redshift, has been detected with the *Fermi* satellite [1].

The details of this effect depend on the Hubble constant, and can therefore be used to measure it [183, 11]. Because it is an optical depth effect, knowledge of the interaction cross-section from basic physics, together with the number density *n*_*p*_ of the interacting photons, allows a length measurement and, assuming knowledge of the redshift of the source, *H*_0_. In practice, the cosmological world model is also needed to determine *n*_*p*_ from observables. From the existing *Fermi* data a value of 72 km s^−1^ Mpc^−1^ is estimated [52] although the errors, dominated by the calculation of the evolution of the extragalactic background light using galaxy luminosity functions and spectral energy distributions, are currently quite large (∼ 10 km s^−1^ Mpc^−1^).

## Local Distance Ladder

### Preliminary remarks

As we have seen, in principle a single object whose spectrum reveals its recession velocity, and whose distance or luminosity is accurately known, gives a measurement of the Hubble constant. In practice, the object must be far enough away for the dominant contribution to the motion to be the velocity associated with the general expansion of the Universe (the “Hubble flow”), as this expansion velocity increases linearly with distance whereas other nuisance velocities, arising from gravitational interaction with nearby matter, do not. For nearby galaxies, motions associated with the potential of the local environment are about 200–300 km s^−1^, requiring us to measure distances corresponding to recession velocities of a few thousand km s^−1^ or greater. These recession velocities correspond to distances of at least a few tens of Mpc.

The Cepheid distance method, used since the original papers by Hubble, has therefore been to measure distances of nearby objects and use this knowledge to calibrate the brightness of more distant objects compared to the nearby ones. This process must be repeated several times in order to bootstrap one’s way out to tens of Mpc, and has been the subject of many reviews and books (see e.g., [176]). The process has a long and tortuous history, with many controversies and false turnings, and which as a by-product included the discovery of a large amount of stellar astrophysics. The astrophysical content of the method is a disadvantage, because errors in our understanding propagate directly into errors in the distance scale and consequently the Hubble constant. The number of steps involved is also a disadvantage, as it allows opportunities for both random and systematic errors to creep into the measurement. It is probably fair to say that some of these errors are still not universally agreed on. The range of recent estimates is in the low seventies of km s^−1^ Mpc^−1^, with the errors having shrunk by a factor of two in the last ten years, and the reasons for the disagreements (in many cases by different analysis of essentially the same data) are often quite complex.

### Basic principle

We first outline the method briefly, before discussing each stage in more detail. Nearby stars have a reliable distance measurement in the form of the parallax effect. This effect arises because the earth’s motion around the sun produces an apparent shift in the position of nearby stars compared to background stars at much greater distances. The shift has a period of a year, and an angular amplitude on the sky of the Earth-Sun distance divided by the distance to the star. The definition of the parsec is the distance which gives a parallax of one arcsecond, and is equivalent to 3.26 light-years, or 3.09 × 10^16^ m. The field of parallax measurement was revolutionised by the *Hipparcos* satellite, which measured thousands of stellar parallax distances, including observations of 223 Galactic Cepheids; of the Cepheids, 26 yielded determinations of reasonable significance [63]. The *Gaia* satellite will increase these by a large factor, probably observing thousands of Galactic Cepheids and giving accurate distances as well as colours and metallicities [225].

Some relatively nearby stars exist in clusters of a few hundred stars known as “open clusters”. These stars can be plotted on a Hertzsprung-Russell diagram of temperature, deduced from their colour together with Wien’s law, against apparent luminosity. Such plots reveal a characteristic sequence, known as the “main sequence” which ranges from red, faint stars to blue, bright stars. This sequence corresponds to the main phase of stellar evolution which stars occupy for most of their lives when they are stably burning hydrogen. In some nearby clusters, notably the Hyades, we have stars all at the same distance and for which parallax effects can give the absolute distance to <1% [159]. In such cases, the main sequence can be calibrated so that we can predict the absolute luminosity of a main-sequence star of a given colour. Applying this to other clusters, a process known as “main sequence fitting”, can also give the absolute distance to these other clusters; the errors involved in this fitting process appear to be of the order of a few percent [5].

The next stage of the bootstrap process is to determine the distance to the nearest objects outside our own Galaxy, the Large and Small Magellanic Clouds. For this we can apply the open-cluster method directly, by observing open clusters in the LMC. Alternatively, we can use calibrators whose true luminosity we know, or can predict from their other properties. Such calibrators must be present in the LMC and also in open clusters (or must be close enough for their parallaxes to be directly measurable).

These calibrators include Mira variables, RR Lyrae stars and Cepheid variable stars, of which Cepheids are intrinsically the most luminous. All of these have variability periods which are correlated with their absolute luminosity (Section 1.1), and in principle the measurement of the distance of a nearby object of any of these types can then be used to determine distances to more distant similar objects simply by observing and comparing the variability periods.

The LMC lies at about 50 kpc, about three orders of magnitude less than that of the distant galaxies of interest for the Hubble constant. However, one class of variable stars, Cepheid variables, can be seen in both the LMC and in galaxies at distances up to 20–30 Mpc. The coming of the Hubble Space Telescope has been vital for this process, as only with the HST can Cepheids be reliably identified and measured in such galaxies.

Even the HST galaxies containing Cepheids are not sufficient to allow the measurement of the universal expansion, because they are not distant enough for the dominant velocity to be the Hubble flow. The final stage is to use galaxies with distances measured with Cepheid variables to calibrate other indicators which can be measured to cosmologically interesting distances. The most promising indicator consists of type Ia supernovae (SNe), which are produced by binary systems in which a giant star is dumping mass on to a white dwarf which has already gone through its evolutionary process and collapsed to an electron-degenerate remnant; at a critical point, the rate and amount of mass dumping is sufficient to trigger a supernova explosion. The physics of the explosion, and hence the observed light-curve of the rise and slow fall, has the same characteristic regardless of distance. Although the absolute luminosity of the explosion is not constant, type Ia supernovae have similar light-curves [163, 8, 209] and in particular there is a very good correlation between the peak brightness and the degree of fading of the supernova 15 days^14^ after peak brightness (a quantity known as Δ*m*_15_ [162, 82]). If SNe Ia can be detected in galaxies with known Cepheid distances, this correlation can be calibrated and used to determine distances to any other galaxy in which a SN Ia is detected. Because of the brightness of supernovae, they can be observed at large distances and hence, finally, a comparison between redshift and distance will give a value of the Hubble constant.

There are alternative indicators which can be used instead of SNe Ia for determination of *H*_0_; all of them rely on the correlation of some easily observable property of galaxies with their luminosity or size, thus allowing them to be used as standard candles or rulers respectively. For example, the edge-on rotation velocity *v* of spiral galaxies scales with luminosity as *L* ∝ *v*^4^, a scaling known as the Tully-Fisher relation [224]. There is an equivalent for elliptical galaxies, known as the Faber-Jackson relation [58]. In practice, more complex combinations of observed properties are often used such as the *D*_*n*_ parameter of [53] and [128], to generate measurable properties of elliptical galaxies which correlate well with luminosity, or the “fundamental plane” [53, 49] between three properties, the average surface brightness within an effective radius^15^ the effective radius *r*_*e*_, and the central stellar velocity dispersion *σ*. Here we can measure surface brightnesses and *σ* and derive a standard ruler in terms of the true *r*_*e*_ which can then be compared with the apparent size of the galaxy.

**Figure 7:**
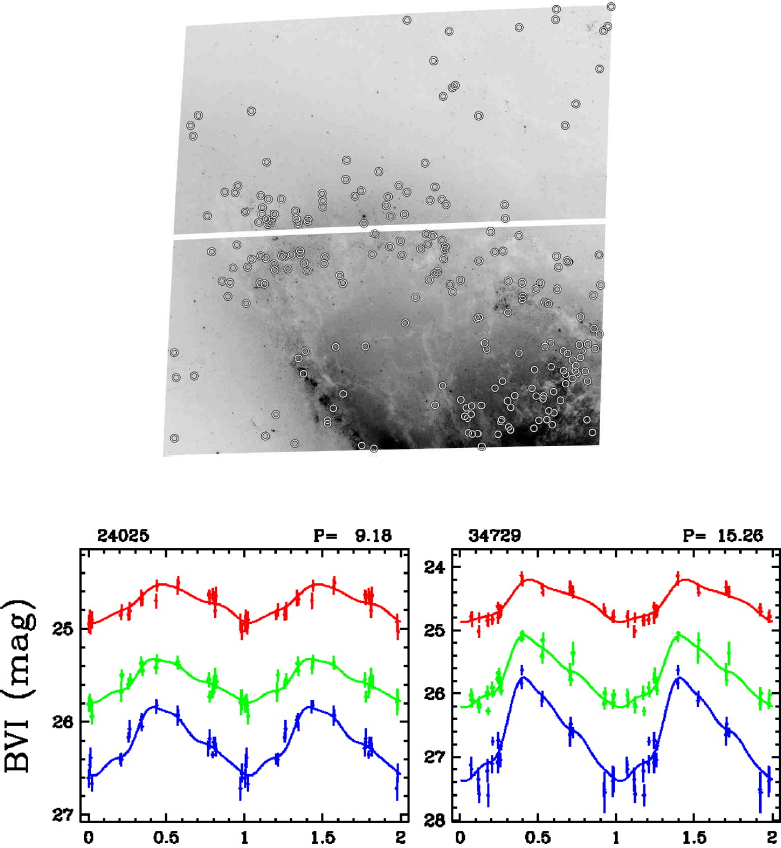
Positions of Cepheid variables in HST/ACS observations of the galaxy NGC 4258. Typical Cepheid lightcurves are shown on the top. Image reproduced with permission from [129]; copyright by AAS.

A somewhat different indicator relies on the fact that the degree to which stars within galaxies are resolved depends on distance, in the sense that closer galaxies have more statistical “bumpiness” in the surface-brightness distribution [219] because of the degree to which Poisson fluctuations in the stellar surface density are visible. This method of surface brightness fluctuation can also be calibrated by Cepheid variables in the nearer galaxies.

### Problems and comments

#### Distance to the LMC

The LMC distance is probably the best-known, and least controversial, part of the distance ladder. Some methods of determination are summarised in [62]; independent calibrations using RR Lyrae variables, Cepheids and open clusters, are consistent with a distance of ∼ 50 kpc. An early measurement, independent of all of the above, was made by [149] using the type II supernova SN 1987A in the LMC. This supernova produced an expanding ring whose angular diameter could be measured using the HST. An absolute size for the ring could also be deduced by monitoring ultraviolet emission lines in the ring and using light travel time arguments, and the distance of 51.2 ± 3.1 kpc followed from comparison of the two. An extension to this light-echo method was proposed in [200] which exploits the fact that the maximum in polarization in scattered light is obtained when the scattering angle is 90°. Hence, if a supernova light echo were observed in polarized light, its distance would be unambiguously calculated by comparing the light-echo time and the angular radius of the polarized ring.

More traditional calibration methods traditionally resulted in distance moduli to the LMC of *μ*° ≃ 18.50 (defined as 5 log *d* − 5, where *d* is the distance in parsecs) corresponding to a distance of ≃ 50 kpc. In particular, developments in the use of standard-candle stars, main sequence fitting and the details of SN 1987A are reviewed by [3] who concludes that *μ*^0^ = 18.50 ± 0.02. This has recently been revised downwards slightly using a more direct calibration using parallax measurements of Galactic Cepheids [14] to calibrate the zero-point of the Cepheid *P-L* relation in the LMC [68]. A value of *μ*^0^ = 18.40 ± 0.01 is found by these authors, corresponding to a distance of 47.9 ± 0.2 kpc. The likely corresponding error in *H*_0_ is well below the level of systematic errors in other parts of the distance ladder. This LMC distance also agrees well with the value needed in order to make the Cepheid distance to NGC 4258 agree with the maser distance ([129], see also Section 4).

#### Cepheid systematics

The details of the calibration of the Cepheid period-luminosity relation have historically caused the most difficulties in the local calibration of the Hubble constant. There are a number of minor effects, which can be estimated and calibrated relatively easy, and a dependence on metallicity which is a systematic problem upon which a lot of effort has been spent and which is now considerably better understood.

One example of a minor difficulty is a selection bias in Cepheid programmes; faint Cepheids are harder to see. Combined with the correlation between luminosity and period, this means that only the brighter short-period Cepheids are seen, and therefore that the *P-L* relation in distant galaxies is made artificially shallow [186] resulting in underestimates of distances. Neglect of this bias can give differences of several percent in the answer, and detailed simulations of it have been carried out by Teerikorpi and collaborators (e.g., [214, 152, 153, 154]). Most authors correct explicitly for this problem — for example, [71] calculate the correction analytically and find a maximum bias of about 3%. Teerikorpi & Paturel suggest that a residual bias may still be present, essentially because the amplitude of variation introduces an additional scatter in brightness at a given period, in addition to the scatter in intrinsic luminosity. How big this bias is is hard to quantify, although it can in principle be eliminated by using only long-period Cepheids at the cost of increases in the random error.

The major systematic difficulty became apparent in studies of the biggest sample of Cepheid variables, which arises from the OGLE microlensing survey of the LMC [227]. Samples of Galactic Cepheids have been studied by many authors [62, 75, 67, 10, 13, 108], and their distances can be calibrated by the methods previously described, or by using lunar-occultation measurements of stellar angular diameters [66] together with stellar temperatures to determine distances by Stefan’s law [236, 9]. Comparison of the *P-L* relations for Galactic and LMC Cepheids, however, show significant dissimilarities. In all three HST colours (*B*, *V*, *I*) the slope of the relations are different, in the sense that Galactic Cepheids are brighter than LMC Cepheids at long periods and are fainter at short periods. The two samples are of equal brightness in *B* at a period of approximately 30 days, and at a period of a little more than 10 days in *I*.^16^

The culprit for this discrepancy is mainly metallicity^17^ differences in the Cepheids, which in turn results from the fact that the LMC is more metal-poor than the Galaxy. Unfortunately, many of the external galaxies which are to be used for distance determination are likely to be similar in metallicity to the Galaxy, but the best local information on Cepheids for calibration purposes comes from the LMC. On average, the Galactic Cepheids tabulated by [81] are of approximately of solar metallicity, whereas those of the LMC are approximately 0.6 dex less metallic. If these two samples are compared with their independently derived distances, a correlation of brightness with metallicity appears with a slope of −0.8 ± 0.3 mag dex^−1^ using only Galactic Cepheids, and −0.27 ± 0.08 mag dex^−1^ using both samples together. This can cause differences of 10–15% in inferred distance if the effect is ignored.

Many areas of historic disagreement can be traced back to how this correction is done. In particular, two different 2005–2006 estimates of 73±4 (statistical) ±5 (systematic) km s^−1^ Mpc^−1^ [170] and 62.3 ± 1.3 (statistical) ±5 (systematic) km s^−1^ Mpc^−1^ [187], both based on the same Cepheid photometry from the HST Key Project [178] and essentially the same Cepheid *P-L* relation for the LMC [218] have their origin mainly in this effect.^18^ One can apply a global correction for metallicity differences between the LMC and the galaxies in which the Cepheids are measured by the HST Key Project [181], or attempt to interpolate between LMC and Galactic *P-L* relations [211] using a period-dependent metallicity correction [187]. The differences in this correction account for the 10–15% difference in the resulting value of *H*_0_.

More recently, a number of different solutions for this problem have been found, which are summarised in the review by [68] and many of which involve getting rid of the intermediate LMC step using other calibrations. [129] use ACS observations of Cepheids in the galaxy NGC 4258, which has a well-determined distance using maser observations (Section 4, [99, 80, 100]), and whose Cepheids have a range of metallicities [242]. Analysis of these Cepheids suggests that the use of a *P-L* relation whose slope varies with metallicity [211, 187] overcorrects at long period. Because of the known maser distance, these Cepheids can then be used both to determine the LMC distance independently [129] and also to calibrate the SNe distance scale and hence determine *H*_0_ [173, 172]. The estimate has been incrementally improved by several methods in the last few years

Values obtained for the Hubble constant using the NGC 4258 calibration are quoted by [174] as 74.8 ± 3.1 km s^−1^ Mpc^−1^, using a value of 7.28 Mpc as the NGC 4258 distance. This was later corrected by [100], who find a distance of 7.60 ± 0.17 (stat) ±0.15 (sys) Mpc using more VLBI epochs, together with better modelling of the masers, which therefore yields a Hubble constant of 72.0 ± 3.0 km s^−1^ Mpc^−1^. Efstathiou [54] has argued for further modifications, with different criteria for rejecting outlying Cepheids; this lowers *H*_0_ to 70.6 ± 3.3 km s^−1^ Mpc^−1^. The alternative distance ladder measurement, using parallax measurements of Galactic Cepheids [14] gives 75.7 ± 2.6 km s^−1^ Mpc^−1^, and using the best available sample of LMC Cepheids observed in the infrared [160] yields 74.4 ± 2.5 km s^−1^ Mpc^−1^. Infrared observations are important because they reduce the potential error involved in extinction corrections. Indeed, the Carnegie Hubble Programme [69] takes this further by using mid-IR observations (at 3.6 *µ*m) of the Benedict et al. Galactic Cepheids with measured parallaxes, thus anchoring the calibration of the mid-IR *P-L* relation in these objects, and obtaining *H*_0_ = 74.3 ± 2.1 km s^−1^ Mpc^−1^. In the mid-IR, as well as smaller extinction corrections, metallicity effects are also generally less. However, arguments for lower values based on different outlier rejection can give a combined estimate for the three different calibrations [54] of 72.5 ± 2.5 km s^−1^ Mpc^−1^.

#### SNe Ia systematics

The calibration of the type Ia supernova distance scale, and hence *H*_0_, is affected by the selection of galaxies used which contain both Cepheids and historical supernovae. Riess et al. [170] make the case for the exclusion of a number of older supernovae from previous samples with measurements on photographic plates. Their exclusion, leaving four calibrators with data judged to be of high quality, has the effect of shrinking the average distances, and hence raising *H*_0_, by a few percent. Freedman et al. [71] included six galaxies including SN 1937C, excluded by [170], but obtained approximately the same value for *H*_0_.

Since SNe Ia occur in galaxies, their brightnesses are likely to be altered by extinction in the host galaxy. This effect can be assessed and, if necessary, corrected for, using information about SNe Ia colours in local SNe. The effect is found to be smaller than other systematics within the distance ladder [172].

Further possible effects include differences in SNe Ia luminosities as a function of environment. Wang et al. [234] used a sample of 109 supernovae to determine a possible effect of metallicity on SNe Ia luminosity, in the sense that supernovae closer to the centre of the galaxy (and hence of higher metallicity) are brighter. They include colour information using the indicator Δ*C*_12_ ≡ (*B* − *V*)_12days_, the *B* − *V* colour at 12 days after maximum, as a means of reducing scatter in the relation between peak luminosity and Δ*m*_15_ which forms the traditional standard candle. Their value of *H*_0_ is, however, quite close to the Key Project value, as they use the four galaxies of [170] to tie the supernova and Cepheid scales together. This closeness indicates that the SNe Ia environment dependence is probably a small effect compared with the systematics associated with Cepheid metallicity.

#### Other methods of establishing the distance scale

In some cases, independent distances to galaxies are available in the form of studies of the tip of the red giant branch. This phenomenon refers to the fact that metal-poor, population II red giant stars have a well-defined cutoff in luminosity which, in the *I*-band, does not vary much with nuisance parameters such as stellar population age. Deep imaging can therefore provide an independent standard candle which can be compared with that of the Cepheids, and in particular with the metallicity of the Cepheids in different galaxies. The result [181] is again that metal-rich Cepheids are brighter, with a quoted slope of −0.24 ± 0.05 mag dex^−1^. This agrees with earlier determinations [111, 107] and is usually adopted when a global correction is applied.

Several different methods have been proposed to bypass some of the early rungs of the distance scale and provide direct measurements of distance to relatively nearby galaxies. Many of these are reviewed in the article by Olling [144].

One of the most promising methods is the use of detached eclipsing binary stars to determine distances directly [147]. In nearby binary stars, where the components can be resolved, the determination of the angular separation, period and radial velocity amplitude immediately yields a distance estimate. In more distant eclipsing binaries in other galaxies, the angular separation cannot be measured directly. However, the light-curve shapes provide information about the orbital period, the ratio of the radius of each star to the orbital separation, and the ratio of the stars’ luminosities. Radial velocity curves can then be used to derive the stellar radii directly. If we can obtain a physical handle on the stellar surface brightness (e.g., by study of the spectral lines) then this, together with knowledge of the stellar radius and of the observed flux received from each star, gives a determination of distance. The DIRECT project [23] has used this method to derive a distance of 964 ± 54 kpc to M33, which is higher than standard distances of 800–850 kpc [70, 124]. It will be interesting to see whether this discrepancy continues after further investigation.

A somewhat related method, but involving rotations of stars around the centre of a distant galaxy, is the method of rotational parallax [161, 145, 144]. Here one observes both the proper motion corresponding to circular rotation, and the radial velocity, of stars within the galaxy. Accurate measurement of the proper motion is difficult and will require observations from future space missions.

## The CMB and Cosmological Estimates of the Distance Scale

### The physics of the anisotropy spectrum and its implications

The physics of stellar distance calibrators is very complicated, because it comes from the era in which the Universe has had time to evolve complicated astrophysics. A large class of alternative approaches to cosmological parameters in general involve going back to an era where astrophysics is relatively simple and linear, the epoch of recombination at which the CMB fluctuations can be studied. Although tests involving the CMB do not directly determine *H*_0_, they provide joint information about *H*_0_ and other cosmological parameters which is improving at a very rapid rate.

In the Universe’s early history, its temperature was high enough to prohibit the formation of atoms, and the Universe was therefore ionized. Approximately 4 × 10^5^ yr after the Big Bang, corresponding to a redshift *z*_rec_ ∼ 1000, the temperature dropped enough to allow the formation of atoms, a point known as “recombination”. For photons, the consequence of recombination was that photons no longer scattered from ionized particles but were free to stream. After recombination, these primordial photons reddened with the expansion of the Universe, forming the cosmic microwave background (CMB) which we observe today as a black-body radiation background at 2.73 K.

In the early Universe, structure existed in the form of small density fluctuations (*δρ*/*ρ* ∼ 0.01) in the photon-baryon fluid. The resulting pressure gradients, together with gravitational restoring forces, drove oscillations, very similar to the acoustic oscillations commonly known as sound waves. Fluctuations prior to recombination could propagate at the relativistic $(c/\sqrt 3)$ sound speed as the Universe expanded. At recombination, the structure was dominated by those oscillation frequencies which had completed a half-integral number of oscillations within the characteristic size of the Universe at recombination;^19^ this pattern became frozen into the photon field which formed the CMB once the photons and baryons decoupled and the sound speed dropped. The process is reviewed in much more detail in [92].

**Figure 8:**
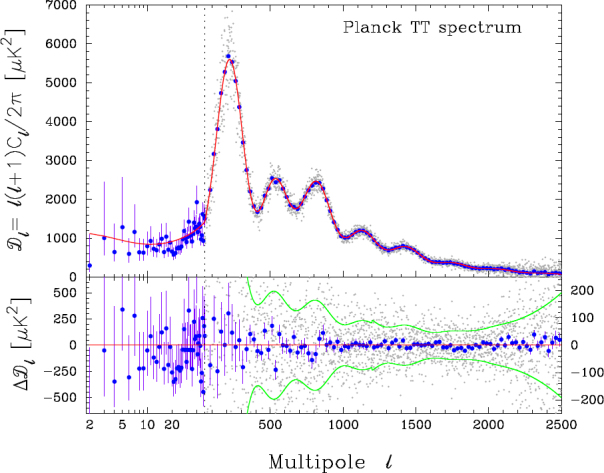
Diagram of the CMB anisotropies, plotted as strength against spatial frequency, from the 2013 *Planck* data. The measured points are shown together with best-fit models. Note the acoustic peaks, the largest of which corresponds to an angular scale of about half a degree. Image reproduced with permission from [2], copyright by ESO.

The resulting “acoustic peaks” dominate the fluctuation spectrum (see Figure 8). Their angular scale is a function of the size of the Universe at the time of recombination, and the angular diameter distance between us and *z*_rec_. Since the angular diameter distance is a function of cosmological parameters, measurement of the positions of the acoustic peaks provides a constraint on cosmo-logical parameters. Specifically, the more closed the spatial geometry of the Universe, the smaller the angular diameter distance for a given redshift, and the larger the characteristic scale of the acoustic peaks. The measurement of the peak position has become a strong constraint in successive observations (in particular BOOMERanG [47], MAXIMA [83], and WMAP, reported in [201] and [202]) and corresponds to an approximately spatially flat Universe in which Ω_m_ + Ω_Λ_ ≃ 1.

But the global geometry of the Universe is not the only property which can be deduced from the fluctuation spectrum.^20^ The peaks are also sensitive to the density of baryons, of total (baryonic + dark) matter, and of vacuum energy (energy associated with the cosmological constant). These densities scale with the square of the Hubble parameter times the corresponding dimensionless densities [see Eq. (5)] and measurement of the acoustic peaks therefore provides information on the Hubble constant, degenerate with other parameters, principally Ω_m_ and Ω_Λ_. The second peak strongly constrains the baryon density, ${\Omega _{\rm{b}}}H_0^2$, and the third peak is sensitive to the total matter density in the form ${\Omega _{\rm{m}}}H_0^2$.

### Degeneracies and implications for *H*_0_

**Figure 9:**
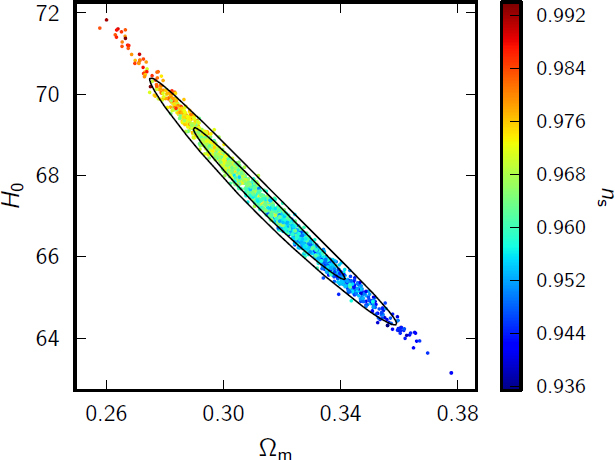
The allowed range of the parameters *H*_0_, Ω_m_, from the 2013 *Planck* data, is shown as a series of points. A flat Universe is assumed, together with information from the *Planck* fluctuation temperature spectrum, CMB lensing information from *Planck*, and WMAP polarization observations. The colour coding reflects different values of *n*_*s*_, the spectral index of scalar perturbations as a function of spatial scale at early times. Image reproduced with permission from [2], copyright by ESO.

Although the CMB observations provide significant information about cosmological parameters, the available data constrain combinations of *H*_0_ with other parameters, and either assumptions or other data must be provided in order to derive the Hubble constant. One possible assumption is that the Universe is exactly flat (i.e., Ω_*k*_ = 0) and the primordial fluctuations have a power law spectrum. In this case measurements of the CMB fluctuation spectrum with the *Wilkinson Anisotropy Probe* (WMAP) satellite [201, 202] and more recently with the *Planck* satellite [2], allow *H*_0_ to be derived. This is because measuring Ω_m_*h*^2^ produces a locus in the Ω_m_: Ω_Λ_ plane which is different from the Ω_m_ + Ω_Λ_ = 1 locus of the flat Universe, and although the tilt of these two lines is not very different, an accurate CMB measurement can localise the intersection enough to give Ω_m_ and *h* separately. The value of *H*_0_ = 73 ± 3 km s^−1^ Mpc^−1^ was derived in this way by WMAP [202] and, using the more accurate spectrum provided by *Planck*, as *H*_0_ = 67.3 ± 1.2 km s^−1^ Mpc^−1^ [2]. In this case, other cosmological parameters can be determined to two and in some cases three significant figures,^21^ Figure 9 shows this in another way, in terms of *H*_0_ as a function of Ω_*m*_ in a flat universe [Ω_*k*_ = 0 in Eq. (10)].

If we do not assume the universe to be exactly flat, as is done in Figure 9, then we obtain a degeneracy with *H*_0_ in the sense that decreasing *H*_0_ increases the total density of the universe (approximately by 0.1 in units of the critical density for a 20 km s^−1^ Mpc^−1^ decrease in *H*_0_). CMB data by themselves, without any further assumptions or extra data, do not supply a significant constraint on *H*_0_ compared to those which are obtainable by other methods. Other observing programmes are, however, available which result in constraints on the Hubble constant together with other parameters, notably Ω_m_, Ω_Λ_ and *w* (the dark energy equation of state parameter defined in Section 1.2); we can either regard *w* as a constant or allow a variation with redshift. We sketch these briefly here; a full review of all programmes addressing cosmic acceleration can be found in the review by Weinberg et al. [235].

The first such supplementary programme is the study of type Ia supernovae, which as we have seen function as standard candles (or at least easily calibratable candles). They therefore determine the luminosity distance *D*_*L*_. Studies of SNe Ia were the first indication that *D*_*L*_ varies with *z* in such a way that an acceleration term, corresponding to a non-zero Ω_Λ_ is required [157, 158, 169], a discovery that won the 2011 Nobel Prize in physics. This determination of luminosity distance gives constraints in the Ω_m_: Ω_Λ_ plane, which are more or less orthogonal to the CMB constraints. Currently, the most complete samples of distant SNe come from SDSS surveys at low redshift (*z* < 0.4) [73, 182, 89, 109], the ESSENCE survey at moderate redshift (0.1 < *z* < 0.78) [135, 238], the SNLS surveys at *z* < 1 [40] and high-redshift (*z* > 0.6) HST surveys [171, 46, 208]. In the future, surveys in the infrared should be capable of extending the redshift range further [175].

The second important programme is the measurement of structure at more recent epochs than the epoch of recombination using the characteristic length scale frozen into the structure of matter at recombination (Section 4.1). This is manifested in the real Universe by an expected preferred correlation length of ∼ 100 Mpc between observed baryon structures, otherwise known as galaxies. These baryon acoustic oscillations (BAOs) measure a standard rod, and constrain the distance measure ${D_V} \equiv {(cz{(1 + z)^2}D_A^2H{(z)^{- 1}})^{1/3}}$ (e.g. [56]). The largest sample available for such studies comes from luminous red galaxies (LRGs) in the Sloan Digital Sky Survey [240]. The expected signal was first found [56] in the form of an increased power in the cross-correlation between galaxies at separations of about 100 Mpc, and corresponds to an effective measurement of angular diameter distance to a redshift *z* ∼ 0.35. Since then, this characteristic distance has been found in other samples at different redshifts, 6dFGS at *z* ≃ 0.1 [15], further SDSS work at *z* = 0.37 [148] and by the BOSS and WiggleZ collaborations at *z* ≃ 0.6 [19, 6]. It has also been observed in studies of the Ly*α* forest [31, 199, 48]. In principle, provided the data are good enough, the BAO can be studied separately in the radial and transverse directions, giving separate constraints on *D*_*A*_ and *H*(*z*) [184, 26] and hence more straightforward and accurate cosmology.

There are a number of other programmes that constrain combinations of cosmological parameters, which can break degeneracies involving *H*_0_. Weak-lensing observations have progressed very substantially over the last decade, after a large programme of quantifying and reducing systematic errors; these observations consist of measuring shapes of large numbers of galaxies in order to extract the small shearing signal produced by matter along the line of sight. The quantity directly probed by such observations is a combination of Ω_m_ and *σ*_8_, the rms density fluctuation at a scale of 8*h*^−1^ Mpc. State-of-the-art surveys include the CFHT survey [85, 110] and SDSS-based surveys [125]. Structure can also be mapped using Lyman-*α* forest observations. The spectra of distant quasars have deep absorption lines corresponding to absorbing matter along the line of sight. The distribution of these lines measures clustering of matter on small scales and thus carries cosmological information (e.g. [226, 133]). Clustering on small scales [215] can be mapped, and the matter power spectrum can be measured, using large samples of galaxies, giving constraints on combinations of *H*_0_, Ω_m_ and *σ*_8_.

#### Combined constraints

As already mentioned, *Planck* observations of the CMB alone are capable of supplying a good constraint on *H*_0_, given three assumptions: the curvature of the Universe,Ω_*k*_ is zero, that dark energy is a cosmological constant (*w* = −1) and that it is independent of redshift (*w* ≠ *w*(*z*)). In general, every other accurate measurement of a combination of cosmological parameters allows one to relax one of the assumptions. For example, if we admit the BAO data together with the CMB, we can allow Ω_*k*_ to be a free parameter [216, 6, 2]. Using earlier WMAP data for the CMB, *H*_0_ is derived to be 69.3 ± 1.6 km s^−1^ Mpc^−1^ [134, 6], which does not change significantly using *Planck* data (68.4 ± 1.0 km s^−1^ Mpc^−1^ [2]); the curvature in each case is tightly constrained (to < 0.01) and consistent with zero. If we introduce supernova data instead of BAO data, we can obtain *w* provided that Ω_*k*_ = 0 [235, 2] and this is found to be consistent with *w* = −1 within errors of about 0.1–0.2 [2].

If we wish to proceed further, we need to introduce additional data to get tight constraints on *H*_0_. The obvious option is to use both BAO and SNe data together with the CMB, which results in *H*_0_ = 68.7 ± 1.9 km s^−1^ Mpc^−1^ [19] and 69.6 ± 1.7 (see Table 4 of [6]) using the WMAP CMB constraints. Such analyses continue to give low errors on *H*_0_ even allowing for a varying *w* in a non-flat universe, although they do use the results from three separate probes to achieve this. Alternatively, extrapolation of the BAO results to *z* = 0 give *H*_0_ directly [55, 15, 235] because the BAO measures a standard ruler, and the lower the redshift, the purer the standard ruler’s dependence on the Hubble constant becomes, independent of other elements in the definition of Hubble parameter such as Ω_*k*_ and *w*. The lowest-redshift BAO measurement is that of the 6dF, which suggests *H*_0_ = 67.0 ± 3.2 km s^−1^ Mpc^−1^ [15].

## Conclusion

Progress over the last few years in determining the Hubble constant to increasing accuracy has been encouraging and rapid. For the first time, in the form of megamaser studies, there is a one-step method available which does not have serious systematics. Simultaneously, gravitational lens time delays, also a one-step method but with a historical problem with systematics due to the mass model, has also made progress due to a combination of better simulations of the environment of the lens galaxies and better use of information which helps to ease the mass degeneracy. The classical Cepheid method has also yielded greatly improved control of systematics, mainly by moving to calibrations based on NGC 4258 and Galactic Cepheids which are much less sensitive to metallicity effects.

Identification of the current “headline” best *H*_0_ distance determinations, by methods involving astrophysical objects, is a somewhat subjective business. However, most such lists are likely to include the following, including the likely update paths:

- Megamasers: 68.0 ± 4.8 km s^−1^ Mpc^−1^ [24]. Further progress will be made by identification and monitoring of additional targets, since the systematics are likely to be well controlled using this method.
- Gravitational lenses: $73.1_{- 3.6}^{+ 2.4}\,{\rm{km}}\,{{\rm{s}}^{- 1}}\,{\rm{Mp}}{{\rm{c}}^{- 1}}$ [206] (best determination with systematics controlled, two lenses), 69±6/4 km s^−1^ Mpc^−1^ [194] (18 lenses, but errors from range of free-form modelling). Progress is likely by careful control of systematics to do with the lens mass model and the surroundings in further objects; a programme (H0LiCOW [204]) is beginning with precisely this objective.
- Cepheid studies: 72.0±3.0 km s^−1^ Mpc^−1^ [174] with corrected NGC 4258 distance from [100]; 75.7± 2.6 km s^−1^ Mpc^−1^ (parallax of Galactic Cepheids) and 74.3± 2.1 km s^−1^ Mpc^−1^ (mid-IR observations) [69]. The Carnegie Cepheid programme is continuing IR observations which should significantly reduce systematics of the method.

In parallel with these developments, the *Planck* satellite has given us much improved constraints on *H*_0_ in combination with other cosmological parameters. The headline *H*_0_ determinations are all from *Planck* in combination with other information, and are:

- For a flat-by-fiat Universe, *H*_0_ = 67.3 ± 1.2 km s^−1^ Mpc^−1^ [2] from *Planck*.
- For a Universe free to curve, *H*_0_ = 68.4 ± 1.0 km s^−1^ Mpc^−1^ [2] using *Planck* together with BAO data.
- Local BAO measurements: *H*_0_ = 67.0±3.2 km s^−1^ Mpc^−1^ [15] using only the well-determined Ω_m_*h*^2^ from the CMB, but independent of other cosmology [15, 19, 6, 148].

There is thus a mild tension between some (but not all) of the astrophysical measurements and the cosmological inferences. There are several ways of looking at this. The first is that a 2.5-*σ* discrepancy is nothing to be afraid of, and indeed is a relief after some of the clumped distributions of published measurements in the past. The second is that one or more methods are now systematics-limited; in other words, the subject is limited by accuracy rather than precision, and that careful attention to underestimated systematics will cause the values to converge in the next few years. Third, it is possible that new physics is involved beyond the variation of the dark energy index *w*. This new physics could, for example, involve the number of relativistic degrees of freedom being greater than the standard value of 3.05, corresponding to three active neutrino contributions [2]; or a scenario in which we are living in a local bubble with a different *H*_0_ [130]. Most instincts would dictate taking these possibilities in this order, unless all of the high-quality astrophysical *H*_0_ values differed from the cosmological ones.

The argument can be turned around, by observing that independent determinations of *H*_0_ can be fed in as constraints to unlock a series of accurate measurements of other cosmological parameters such as *w*. This point has been made a number of times, in particular by Hu [91], Linder [126] and Suyu et al. [207]; the dark energy figure of merit, which measures the *P* − *ρ* dependence of dark energy and its redshift evolution, can be be improved by large factors using such independent measurements. Such measurements are usually extremely cheap in observing time (and financially) compared to other dark energy programmes. They will, however, require 1% determinations of *H*_0_, given the current state of play in cosmology. This is not impossible, and should be reachable with care quite soon.

## Footnotes

1 See [105] and references therein, e.g., [27], for discussion of the details of the interpretation of redshift in an expanding Universe. The first level of sophistication involves maintaining the GR principle that space is locally Minkowskian, so that in a small region of space all effects must reduce to SR (for instance, in Peacock’s [155] example, the expansion of the Universe does not imply that a long-lived human will grow to four metres tall in the next 10^10^ years). Redshift can be thought of as a series of transformations in photon wavelengths between an infinite succession of closely-separated observers, resulting in an overall wavelength shift between two observers with a finite separation and therefore an associated “velocity”. The second level of sophistication is to ask what this velocity actually represents. [105] calculates the ratio of photon wavelength shifts between pairs of fundamental observers to the shifts in their proper separation in the presence of arbitrary gravitational fields, and shows that this ratio only corresponds to the purely dynamical result if the gravitational tide is constant.

2 This is known in the literature as the “instability strip” and is almost, but not quite, parallel to the luminosity axis on the H-R diagram. In normal stars, any compression of the star, and the associated rise in temperature, results in a decrease in opacity; the resulting escape of photons produces expansion and cooling. For stars in the instability strip, a layer of partially ionized He close to the surface causes opacity to rise instead of falling with an increase in temperature, producing a degree of positive feedback and consequently oscillations. The instability strip has a finite width, which causes a small degree of dispersion in period-luminosity correlations among Cepheids.

3 There are numerous subtle and less-subtle biases in distance measurement; see [213] for a blow-by-blow account. The simplest bias, the “classical” Malmquist bias, arises because, in any population of objects with a distribution in intrinsic luminosity, only the brighter members of the population will be seen at large distances. The result is that the inferred average luminosity is greater than the true luminosity, biasing distance measurements towards the systematically short. The Behr bias [12] from 1951 is a distance-dependent version of the Malmquist bias, namely that at higher distances, increasingly bright galaxies will be missing from samples. This leads to an overestimate of the average brightness of the standard candle which becomes worse at higher distance.

4 Cepheids come in two flavours: type I and type II, corresponding to population I and II stars. Population II stars are an earlier metal-poor generation of stars, which formed after the hypothetical, truly primordial Population III stars, but before later-generation Population I stars like the Sun which contain significant extra amounts of elements other than hydrogen and helium due to enrichment of the ISM by supernovae in the meantime. The name “Cepheid” derives from the fact that the star *δ* Cephei was the first to be identified (by Goodricke in 1784). Population II Cepheids are sometimes known as W Virginis stars, after their prototype, W Vir, and a W Vir star is typically a factor of 3 fainter than a classical Cepheid of the same period.

5 The conclusion of the latter, that based on median statistics of the Huchra compilation, *H*_0_ = 67 ± 2 km s^−1^ Mpc^−1^, is slightly scary in retrospect given the *Planck* value of 67.2 ± 1.3 km s^−1^ Mpc^−1^ for a flat Universe [2].

6 Historically, the Hubble constant has often been quoted as *H*_0_ = 100*h* km s^−^1 Mpc^−1^, as a way of maintaining agnosticism in an era where observations allowed a wide range in *h*. This is largely disappearing, but papers using *h* can be hard to interpret [42]

7 For example, one topic that may merit more than a footnote in the future is the study of cosmology using gravitational waves. In particular, a coalescing binary system consisting of two neutron stars produces gravitational waves, and under those circumstances the measurement of the amplitude and frequency of the waves determines the distance to the object independently of the stellar masses [193]. This was studied in more detail by [36] and extended to more massive black-hole systems [90, 45]. More massive coalescing signals produce lower-frequency gravitational-wave signals, which can be detected with the proposed LISA space-based interferometer (http://lisa.nasa.gov/documentation.html). The major difficulty is obtaining the redshift measurement to go with the distance estimate, since the galaxy in which the coalescence event has taken place must be identified. Given this, however, the precision of the *H*_0_ measurement is limited only by weak gravitational lensing along the line of sight, and even this is reducible by observations of multiple systems or detailed investigations of matter along the line of sight. *H*_0_ determinations to ∼ 2% should be possible, but depend on the launch of LISA or a similar mission. This is an event that is probably decades away, although a pathfinder mission to test some of the technology is due for launch in 2015.

8 Strictly speaking, provided we ignore effects to do with curvature of the Universe.

9 An isothermal model is one in which the projected surface mass density decreases as 1/*r*. An isothermal galaxy will have a flat rotation curve, as is observed in many galaxies.

10 Essentially all radio time delays have come from the VLA, although monitoring programmes with MERLIN have also been attempted.

11 The redshifts of the lens and source also need to be known, as does the position of the centre of the lens galaxy; this measurement is not always a trivial proposition [241].

12 As discussed extensively in [113, 117], this is not a global degeneracy, but arises because the lensed images tell you about the mass distribution in the annulus centred on the galaxy and with inner and outer radii defined by the inner and outer images. Kochanek [113] derives detailed expressions for the time delay in terms of the central underlying and controlling parameter, the surface density in this annulus [76].

13 The programme, known as H0LiCOW, is now continuing in order to measure time delays, improve models and derive further *H*_0_ values for more lenses.

14 Because of the expansion of the Universe, there is a time dilation of a factor (1 + *z*)^−1^ which must be applied to timescales measured at cosmological distances before these are used for such comparisons.

15 The effective radius is the radius from within which half the galaxy’s light is emitted.

16 Nearly all Cepheids measured in galaxies containing SN Ia have periods > 20 days, so the usual sense of the effect is that Galactic Cepheids of a given period are brighter than LMC Cepheids.

17 Here, as elsewhere in astronomy, the term “metals” is used to refer to any element heavier than helium. Metallicity is usually quoted as 12+log(O/H), where O and H are the abundances of oxygen and hydrogen.

18 The details are discussed in more detail in an earlier version of this review [102].

19 This characteristic size is about 4 × 10^5^ light years at recombination, corresponding to an angular scale of about 1° on the sky. The fact that the CMB is homogeneous on scales much larger than this is an illustration of the “horizon problem” discussed in Section 1.2, and which inflation may solve.

20 See http://background.uchicago.edu/∼whu/intermediate/intermediate.html for a much longer exposition and tutorial on all these areas.

21 See Table 2 of [2] for a full list.
